# DNA Methylation and Epigenetic Events Underlying Renal Cell Carcinomas

**DOI:** 10.7759/cureus.30743

**Published:** 2022-10-27

**Authors:** Imrana Tanvir, Amber Hassan, Fatma Albeladi

**Affiliations:** 1 Pathology, King Abdulaziz University, Jeddah, SAU; 2 System Medicine (Molecular Oncology), European School of Molecular Medicine (SEMM) University of Milan, Milan, ITA; 3 System Medicine (Molecular Oncology), Translational Neuroscience Lab, CEINGE Biotecnologie Avanzate, Naples, ITA; 4 Nephrology, King Abdulaziz University, Jeddah, SAU

**Keywords:** genetic and epigenetic analysis technologies, dna methylation, von hippel-lindau, kidney cancer, malignant cells, epigenetic driver genes, dnmts, rcc, methylation

## Abstract

Renal cell carcinoma (RCC) refers to a group of tumors that develop from the epithelium of the kidney tubes, including clear cell RCC, papillary RCC, and chromophobe RCC. Most clear cell renal carcinomas have a large histologic subtype, genetic or epigenetic von Hippel-Lindau (VHL). A comprehensive analysis of the genetic modification genome suggested that chromosome 3p loss and chromosome gains 5q and 7 may be significant copy defects in the development of clear RCC. A more potent RCC may develop if chromosome 1p, 4, 9, 13q, or 14q is also lost. Renal carcinogenesis is not associated with chronic inflammation or histological changes. However, if regional hypermethylation of DNA in CpG C-type islands has already accumulated in cancer-free kidney tissue, it implies that the presence of malignant kidney lesions may also be detected by modified DNA methylation. Modification of DNA methylation in cancerous kidney tissue may advance kidney tissue to epigenetic mutations and genes, leading to more serious cancers and even determining a patient's outcome. The genetic and epigenetic profile provides accurate predictors for patients with kidney cancer. New genetic and epigenetic analysis technologies will help to speed up the identification of vital cells for kidney cancer prevention, diagnosis, and treatment.

## Introduction and background

Cancer, as a genetic disorder, is associated with epigenetic abnormalities. It is clear that epigenetic disruption caused by the microenvironment is important in developing neoplasia [1]. Changes in gene expression that occur without altering DNA sequences and are powerful enough to control genetic variation are referred to as epigenetics [2]. The major mechanisms responsible for epigenetic regulation are DNA methylation, histone modification, and posttranscriptional regulation that does not encode RNA, also known as microRNAs [3]. These mechanisms are critical components of normal cell development and growth, and their modification contributes to plastic phenotypes [4]. Renal cell carcinoma (RCC) is one of the top 10 most common diseases, accounting for 2-3% of all adult-related diseases and over 100,000 deaths worldwide each year [5].

The most common type of RCC is clear cell RCC (ccRCC), which accounts for 75% of all RCC cases [6]. Recent advances in DNA sequencing technologies benefit diagnostic and clinical sites [7]. In addition to genetic mutations, DNA methylation has been identified in all cancers, including ccRCC. The role of other epigenetic mechanisms in tumorigenesis has not been thoroughly investigated [8]. Epigenetic events may promote tumorigenesis and determine tumor progression. As a result, they can be used to track treatment response and treatment modalities [9]. Furthermore, epigenetic modification can be reversed and altered. Mapping the differences between normal tissue and tumor cells will thus provide new information that can be used to identify functional regions or genes ("epigenetic driver genes") that respond to epigenetic changes and ultimately promote tumorigenesis [10].

 Emerging evidence suggests that modifying our body through exercise or a variety of foods such as ketogenic diets, low-carbohydrate diets, fasting, or exercise can alter the concentration of various metabolites, some of which can alter the function of proteins that cause epigenetic changes [3,4]. These epigenetic modifications appear to regulate important genetic networks that mediate the body's processes associated with the beneficial effects of these diets and represent a simple and logical way to prevent or even cure these diseases [11].

DNA methylation may be the most studied epigenetic marker among the epigenetic components. DNA methylation is a type of post-genetic mutation that occurs in the cytosine sequence of 5'-C-phosphate-G-3' (CpG) dinucleotide, in which the methyl group S-adenylmethionine is exchanged with cytosine [12]. Additional methyl groups result in the crossroads, and when DNA is symmetrically methylated, methyl groups promote mutations in DNA structure [13]. There is mounting evidence that epigenetic changes and genetic mutations that occur during tumorigenesis are linked [14]. However, these changes usually occur independently [15]. In the case of tumorigenesis, recent research has revealed that DNA methylation mutations are linked to a variety of human diseases, including cancer [16].

The main goal of this review is to provide an overview of the role of DNA methylation in the pathology of RCC. We will first discuss the relationship between epigenetics and DNA methylation before delving into recent developments in DNA methylation research in RCC and the role of DNA methylation in therapeutic approaches. Overall, these findings point to a novel method for identifying the gene for an epigenetic driver, the intended therapeutic target of a ccRCC treatment strategy, including self-medication.

## Review

DNA methylation and histone modification

Epigenetics is the study of phenotypic mutations that do not involve DNA sequencing or just genes. It affects the function of genes by influencing their cellular and physiological phenotype expression [17]. The variety of environmental factors that are part of normal human development can be their influencer. Thus, to define epigenetics, these mutations must be inherited [18]. Epigenetics initiates the opening/closing of genes to produce proteins. As mentioned earlier, human cells are involved in epigenetic changes throughout their lives. Indeed, identical twins with the same genetic makeup accumulate different epigenetic patterns depending on their environmental factors, such as diet, tobacco, or exercise [19]. DNA methylation, histone modification, and non-coded RNA action are all major epigenetic pathways [20]. Among these, DNA methylation is the most extensively researched epigenetic insignia, with numerous studies examining its relationship to disease development [21]. DNA methylation is a reversible process that introduces methyl groups (-CH3) into cytosine in CpG nucleotides (5'-cytosine phosphate-guanosine-3'), converting this cytosine into five methylcytosines (5mC). This process changes the balance and accessibility of DNA, as well as controls genetic expression. DNA methylation is carried out by specific enzymes known as de novo DNA methyltransferases (DNMTs) and takes place at the expense of ATP and S-adenosylmethionine as methyl group contributors [22]. DNMTs are expressed in tissue and cell-specific mechanisms during neuronal development and in the adult brain, including active neurogenesis and adult stem cell niches, where they participate in neuronal plasticity and survival [23]. After methylation is complete, proteins from the methyl-CpG-binding (MBD) family bind to methylated loci to promote the registration of histone modulatory mutations, indicating synergistic mutations for multiple epigenetic markers [24]. Hydroxymetylation (5hmC) is another important mechanism related to DNA methylation and is another epigenetic mechanism that converts five methylcytosines and adds a hydroxymethyl group. Hydroxymethylation is involved in important processes such as genetic control and isolation [25].

Epigenetic marker is very common in cancer cells representing both a central stage in the demethylation process and an important epigenetic marker in tumorigenesis [26]. Although DNA demethylases such as activation-induced cytidine deaminase and the DNA demethylation function of TET1 (a member of TETs) have been identified, the process of DNA demethylation and the enzymes that make up this reaction remain unknown [27]. Given the growing evidence that DNA methylation plays an important role in common diseases, researchers have attempted to use DNA methylation as a biomarker to detect epigenetic mutations linked to disease status. The biological patterns associated with cancer progression are determined by the global balance of DNA methylation, demethylation, and hydroxymethylation in cancer [28].

Cancer epigenetics modifications

**Figure 1:**
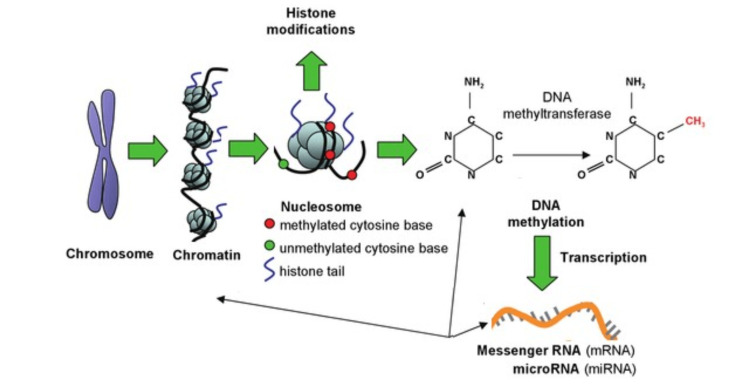
Schematic diagram of epigenetics modification In cancer, damage (genetic mutations and epigenetics) leads to significant changes. DNA methylation, histone modification, and RNA-associated mutation work together to stem the tide of genetics. Adapted from:  Relton and Davey Smith, 2010 [34]

Cancer epigenetics deals with mutations in the DNA of malignant cells and excludes mutations in DNA sequences [17]. Loss of gene expression occurs more often in the context of textual silence influenced by epigenetic promoters, i.e. hypermethylation of CpG islands, than in genetic mutation [29]. In the study of colorectal carcinoma, Vogelstein et al. found that there was no methylation in the surrounding mucosa and 600 to 800 in CpG islands that were more methylated in the intestinal promoters compared to normal mucosa near the tumor [30]. Therefore, they have found that it is very promising to deceive epigenetic mutations. Therefore, controlling various epigenetic factors can influence the prevention, diagnosis, treatment, and prognosis of cancer. Over time, various cancers have been linked to a variety of influential epigenetic factors that, if we scientists can control them, such as tumor-suppressing genes, histone mutations, changes in DNA binding proteins, and regeneration of oncogenes due to mutations [31]. Methylation of CpG islands can affect tissue [32]. Several epigenetic therapies are now used in today's world. So far, we have come to appreciate the value of epigenetics in the development of a particular living thing. From a single cell to an embryo that grows muscle cells, nerve cells, liver cells, or any other type of cell. How a cell type is determined is controlled by a specific group of open genes? It is therefore the epigenetic factors that influence which genes are activated and do not work [1]. In cancer, damage (genetic mutations) and (epigenetics) leads to significant changes." So far, the three systems work together to stem the tide of genetics. These three include DNA methylation, histone modification, and RNA-associated mutation [33] (Figure 1).

DNA methylation in cancer

The methyl-CpG-binding domain (MBD) "epigenome readers" methyl-CpG binding protein 2 (MeCP2) and methyl-CpG-binding domain proteins 1-4 (MBD1-4) can detect DNA methylation [22]. Epigenetics plays a role in the development of neoplasia in mammalian systems, including initiation, proliferation, invasion, and metastasis. Rare DNA methylation patterns are frequently associated with genome-wide hypomethylation and promoters with a specific site of CpG hypermethylation. Epigenetic mutations, which are linked to tumor progression, are caused by different cell types [34]. The hypermethylation promoter activates genes involved in cellular processes such as DNA repair, gene repair 1 (hMLH1), O6-methylguanine-DNA methyltransferase (MGMT), Werner syndrome, as RecQ helicase (WRN), breast 1 WIF-1, and SFRP1. Cadherin 1 (CDH1), CDH13, and PCDH10 are found in metastasis [22, 35].

Among others, the hormone response ESR1, ESR2 in the p53 network [p14ARF, p7], and HIC-1 promote tumor cell growth while increasing genetic instability and aggression [36]. Oncogenes are frequently associated with hypomethylation [37]. C-Myc, an oncogene transcription factor, is one of the most commonly hypomethylated genes found in cancer. Hypomethylation in some promoters can cause oncogenes to express in the opposite direction, resulting in print loss (LOI). Insulin-like growth factor 2 (IGF2) is the most common cause of LOI due to hypomethylation, and it has been linked to a variety of tumors, including breast, liver, lung, and colon cancer [38]. S100 calcium-binding protein P (S100P) for pancreatic cancer [39], synuclein gamma (SNCG) for breast and ovarian cancer [38], melanoma-related gene (MAG, E) [40], and dipeptidyl peptidase 6 for dipeptidyl peptidase 6 (DPP6) in melanoma are well-studied examples of hypomethylated genes in cancer [41]. The modification reduces heterochromatin binding to the G2 cell cycle and impairs DNA methyltransferase activity, resulting in extensive hypomethylation and local hypermethylation, resulting in abnormal methylation patterns that may explain its complex role in cancer progression (39). Recent research has found that the rate of histone conversion predicts genetic expression. Acetylation loss promotes the overexpression or conversion of histone deacetylases (HDACs) to various types of tumors [42]. In renal carcinomas, inactive mutations in histone methyltransferase SETD2, histone demethylase UTX, and JARID1C have been described [43]. miRNA expression patterns appear to indicate a dangerous condition because abnormal cell proliferation is a sign of human cancer. Other types of tumors have been found to have altered manifestations of other miRNAs [43, 44]. Let-7 is one of the most well-studied cancerous miRNA families. The let-7 function has been altered in a variety of cancers, including those of the head and neck, lungs, colon, rectum, and ovary. It is an extremely effective tumor suppressor miRNA [45]. miRNA-145 is a well-known tumor suppressor miRNA that is downregulated in the majority of human colds due to incorrect DNA methylation of its promoter and/or p53 mutations. Significantly, the miRNA-29 family can directly control the expression of DNMTs, so down-regulation of this miRNA family in small lung cancers leads to increased DNMT3A and 3B expression, which leads to global genomic hypermethylation and methylation-in silencing of tumor-suppressing genes like FHIT and WWOX [46] (Table 1).

**Table 1:** Methylated genes in cancer cellular pathways MDS: myelodysplastic syndrome

Pathways	Methylated Genes	Cancer Pathways
Growth Signal Autonomy	RASSFIA, SOCS1	Lung, Bladder, Ovarian, Breast, Lymphoma, MDS, Gastric
Insensitivity to anti-Growth Signals	p15, p16	Melanoma, Lymphoma, Bladder
Evading Apoptosis	DAPK	Lymphoma
Tumor Invasion and Metastasis	CDH1, TIMP3	Gastrointestinal, Esophagus
Sustained Angiogenesis	THBS1	Lymphoma, Neuroblastoma, Endometrial
Genomic Instability	MGMT CHFR MLH1 LMNA	Lymphoma, colon Gastric Colon Lymphoma

When a methyl group (CH3) is added to or removed from DNA, this is referred to as methylation. These mutations result in genetic mutations, which promote growth. overmethylation of DNA typically involves inserting a methyl group into a 5-carbon cytosine ring, yielding 5-methylcytosine [47]. This results in a massive downpour of DNA and the inhibition of transcription. Cancer cells frequently exhibit DNA hypomethylation, which promotes tumorigenesis [48]. Till today. according to the literature, 24 metastasis is known to be epigenetically regulated by DNA hypomethylation. The association between promoter hypomethylation and increased expression of the protease-encoding urokinase plasminogen activator gene (PLAU) and progressive breast and prostate cancer has been established [49].

DNA methylation and cancer metastasis

Cancer metastasis involves stages of local invasion and proliferation. This is influenced by the oncogenic suppressive transcription factor (TFs) that regulate tumor microenvironment features [50]. DNA methylation disrupts the network and affects metastasis. By focusing on recent research on the control of metastasis, we as scientists can use therapeutic by identifying these controls [51]. Epigenetics leading to alteration influences cancer metastasis, which is a real challenge for cancer treatment. Experimental systems show that cancer cells store and develop specific signaling pathways needed for metastasis, but many of these mechanisms are unknown to researchers [47]. New evidence suggests that oncogenic signals that alter transcriptional mutations automatically lead to metastasis symptoms resulting in onset and progression [52]. To fully understand the causes of metastasis, molecular defining mechanisms remain a challenge. Studies show that epigenetics controls the blood vessels associated with a tumor [53]. Various, unstable, continuous comparable factors are associated with malignant tumor cell genome leading to metastatic rupture [54]. Many known epigenetic factors such as inflammation, hypoxia, growth factors, etc., can have genetic effects such as oncogene expression and genetic loss that suppresses the tumor [54,55]. These changes affecting the stage and site in regulating angiogenesis are also dependent on angiogenesis [56]. These mutations, in turn, lead to the ability to differentiate metastatic cancer cells, sometimes from the same patient [57]. How these genetic and epigenetic events are related to the growth and metastasis of cancer cells is yet to be studied in the future, which can lead to the effective use of anti-angiogenesis drugs.

Tumor-related genes and their role in renal carcinogenesis

Although RCC classification is largely based on histology, the World Health Organization (WHO) classification has introduced genetic mutations as a sign of certain types of histological subtypes of RCC, for example, cell RCC is characterized by chromosome 3p loss and VHL gene dysfunction at 3p25.3 due to mutation or DNA methylation around the promoter region [5]. The VHL product is a multifunctional 3-kDa protein with a well-documented role in substrate recognition by the E3-ubiquitin ligase complex [58]. This complex is best known for detecting hypoxia-inducible (HIFs) polyubiquitination and proteasome degeneration [59]. Under hypoxic conditions, HIF-1 alpha and HIF-2 alpha bind together to form HIF-1beta heterodimers, which then transmit to the nucleus, where they stimulate downstream gene expression, including vascular endothelial growth factor (VEGF) [60]. The absence of wild VHL promotes incorrect activation of targeted genes, which contributes to tumorigenesis [61]. Furthermore, the VHL protein has independent functions in HIF-1alpha and HIF-2alpha and is thought to be required for tumor suppression, cell-matrix integration, microtubule dynamics control, apoptosis control, and possibly TTP53 protein stability [62].

**Figure 2:**
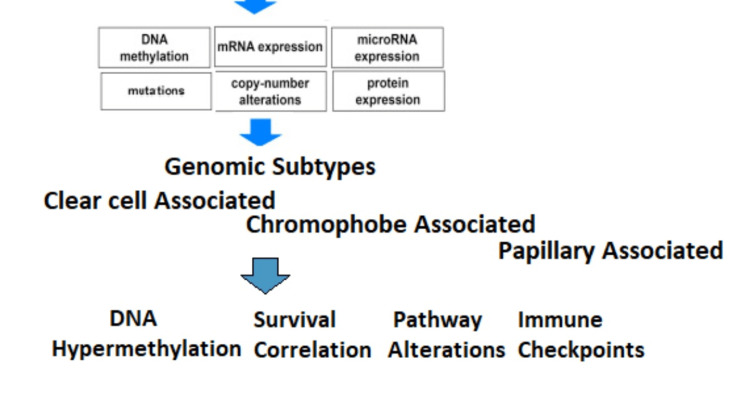
Genomics taxonomy of renal cell carcinoma

Type 1 papillary RCC develops in patients with genetic mutations who benefit from mesenchymal epithelial transition (MET) genetics. Transmembrane receptor tyrosine kinase is incorporated into MET's ligand, hepatocyte growth factor (HGF). MET activation of HGF causes tyrosine kinase activity, which facilitates several transduction cascades leading to many cellular processes such as mitogenesis and migration [63]. However, the incidence of MET conversion in sporadic papillary RCC is low (around 10%). Type 2 papillary RCC is caused by viral mutations in fumarate hydratase (FH) [64]. VHL recognition of HIF necessitates hydroxylation by HIF prolyl hydroxylase (HPH), which FH activates. Because of HPH dysfunction, FH mutation promotes tumorigenesis by accumulating HIF protein [65]( Figure 2).

The excessive c-kit function (KIT) expression occurs in the chromophobe RCC, in contrast to the genetic modification of KIT: KIT is a type III receptor tyrosine kinase that participates in cell signaling [66]. When KIT binds to a ligand, such as a stem cell factor, it is usually phosphorylated. This initiates a phosphorylation cascade, which ultimately activates various aspects of transcription [67]. Apoptosis, cell division, proliferation, chemotaxis, and cell adhesion are all regulated by this activation. Although BHD gene mutations, including folliculin, have been found in 80% of BHD strains, chromophobe RCC mutations are much rarer [68]. TSC has been linked to germline TSC1 (9q34) hamartin encoding or TSC2 (16p13.3) encoding tuberin mutations and affected patients have an increased risk of developing kidney tumors such as ccRCC, papillary RCC, and chromophobe RCC [69]. The TSC1 / TSC2 protein complex inhibits the rapamycin target oapamycin (mTOR) and is involved in signaling pathways that control cell growth. Although the TSc2 gene Eker-infected mouse model has a highly inherited cancer [70], the role of TSC1 and TSC2 in RCC in some individuals is unknown.

RAS, v-RAF murine sarcoma viral oncogene B1 (BRAF) [71], TP53 [72], retinoblastoma (RB) [73], cyclin-dependent kinase inhibitor 2A (CDKN2A) [74], phosphoinositide-3 -kinase, catalytic alpha polypeptide (PIK3CA) [75], phosphatase and tensin homolog (PTEN) [76], epidermal growth factor receptor (EGFR) [77], Somatic truncating mutations in the neurofibromin 2 (NF2) gene, which encodes marlin proteins such as ERM (ezrin, radixin, moesin) family members that connect cytoskeletal components and cell membranes [78], have recently been reported in RCC cells clear. It has been suggested that in the absence of explicit RCC cell samples with VHL-converted NF2 mutations, somatic NF2 mutations may account for half of the cases in this subclinical [79, 80] (Table 2).

**Table 2:** DNA methylation alterations in human cancers

DNA Methyltransferase	Function	Alterations	Cancer Type
DNMT 1	Maintenance of Methylation	Upregulation, Mutation	Ovarian and Colorectal Cancer
DNMT3a	De novo Methylation during Embryogenesis	Upregulation	Breast, Oral Squamous cell, Ovarian, and Colorectal Cancer
DNMT3b	De novo Methylation during Embryogenesis Repeat Methylation Repression	Upregulation	Breast, Hepatocellular, and Colorectal Cancer

Genetic clustering of ccRCCs

Since the genetic background of RCCs is not fully understood, array-comparative genomic hybridization (CGH) is being analyzed and modified using a customized bacterial artificial chromosome (BAC) array (MCG Whole Genome Array-4500) [81]. The RCC is usually surrounded by a fibrous and well-designed cortex, with no fibrous stroma between cancer cells [82]. Current genome-wide analysis has shown that chromosome 3p loss and 5q and 7 gain significant copy defects in the development of ccRCC cells, regardless of genetic interaction [83]. Further loss of chromosomes 1p, 4, 9, 13q, or 14q may increase the risk of cluster BTG [84]. There is now compelling evidence that genetic global expression profiling can identify cancer subtypes based on underlying heterogeneity in mutation, cell division, or cell types [84]. Recent research, for example, has revealed that two types of breast cancer (BRCA1 and 2) have distinct genetic profiles [85], implying that differences in gene expression are caused by differences in genetic modification. Another study found that the gene expression profiles of hepatocellular carcinoma patients differed depending on whether they were hepatitis B or hepatitis C virus-positive [86], implying that the tumorigenesis process influences the genetic profile [87]. Genetic profiles can help with a more accurate and objective cancer diagnosis, disease speculation, and treatment response. A recent study of large B-cell lymphoma tumors revealed very different survival prospects based on abstract genetic profiles, so patient samples with long-term follow-up information are required to evaluate the predictive value of specific gene expression profiles [88]. The same research into other deadly diseases is expected, but it remains difficult because it requires both proper maintenance of used tissue and long-term patient follow-up data.

Clinical implications of DNA methylation as a marker of RCC disease

Clinically, most cases of RCC are less obvious and are now diagnosed as a result of the unintentional use of abdominal computed tomography (CT), ultrasound (US), and magnetic resonance tomography (MRT) for other medical reasons [89]. Early detection is critical to effective cancer treatment. Meanwhile, 30% of RCC patients have metastases at the time of diagnosis, and another 30-50% will have metastases during follow-up, even if major surgery has been performed previously [90]. If metastases are present in the diagnosis, the five-year survival rate may be less than 10-15%, whereas patients with the local disease have a five-year survival rate of up to 95% [91]. As a result, there is an urgent need to develop new molecular biomarkers for the early detection of ccRCC and the identification of patients at high risk of progression. During the onset and progression of cancer, common epigenetic processes, such as genome-wide mutations in DNA methylation patterns, are disrupted [92].

 Hypermethylation of CpG islands is common in a variety of cancers, including kidney cancer, and is frequently associated with tumor-suppressor gene mutations and signatory mechanisms [93]. During renal cell carcinogenesis, epigenetic control mutations are observed, resulting in numerous changes in DNA methylation [94]. Because abnormal DNA methylation is one of the earliest cell mutations in cancer, these mutations can be useful in disease diagnosis and/or prognosis [95]. Despite their potential, no accurate or predictable RCC DNA methylation biomarker has yet reached the clinic. Methylated DNA found in urological tumors, particularly RCC, can be easily detected in urine samples, allowing for the development of invasive, non-invasive cell testing [96]. Furthermore, ccRCC is a fatal disease with high intra-tumor and inter-tumor heterogeneity, making diagnosis and prediction difficult [97]. DNA methylation in urine aggravates this condition, providing a more accurate representation of tumor heterogeneity than a tissue sample [98]. Furthermore, due to the ease with which samples can be replicated, urine-based biological symptoms can be observed on a regular basis in at-risk patients, allowing for early detection of tumors or tracking the progression of cancer in real-time [99]. A number of DNA methylation biomarkers, including ZNF677, FBN2, PCDH8, TFAP2B, TAC1, and FLRT2, were found in kidney tissue and urine samples from patients with ccRCC and provided significant clinical assistance and promising power that does not exist in detection and prediction of invading ccRCC [100, 101].

**Figure 3:**
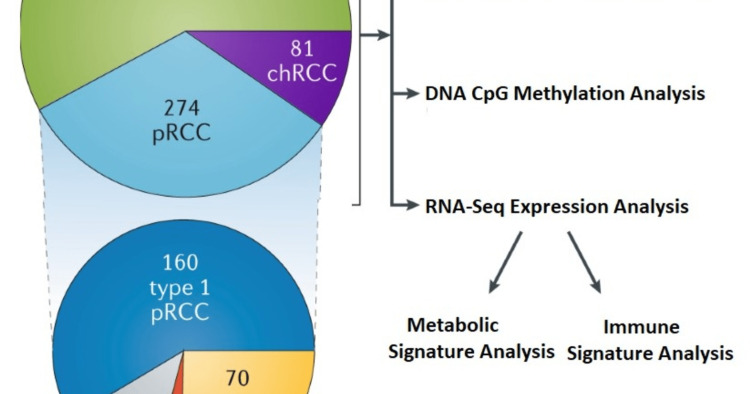
The Cancer Genome Atlas of renal cell carcinoma: findings and clinical implications The Genomic Atlas Cancer Analysis confirmed a few well-known aspects of RCC while also expanding our understanding of many other factors, such as survival biomarkers pRCC: papillary renal cell carcinoma; chRCC: chromophore renal cell carcinoma; CpG: 5'—C—phosphate—G—3' Adapted From: Linehan and Ricketts, 2019 [106] with permission from Springer Nature

The Genomic Atlas Cancer Analysis (TCGA) confirmed a few well-known aspects of RCC while also expanding our understanding of many other factors, such as survival biomarkers [102]. The findings extend the correlation of CDKN2A loss with decreased survival in ccRCC and papillary RCC (pRCC) to chromophore RCC (chRCC) and demonstrate that mutation metabolism is associated with negative predictors in patients with ccRCC or metabolic-separated chRCC [103]. Furthermore, a thorough examination of known genetic combinations as well as novel TFE3 and TFEB in RCC tumors with varying histological features highlighted the importance of considering RCC family MiT transfers in patients of all ages [104]. Studies confirmed these findings by detecting melanocyte inducing transcription factor (MITF) genetic mutations in adult patients [105](Figure 3).

Epigenetic changes as targets for cancer therapy

Epigenetic methods as a new treatment have aroused much interest in recent research over the past few decades. Epigenetic mutations can initiate disease and may predict clinical outcomes [107]. Recent genomic studies have linked ccRCC to the conversion of chromatin-converting enzymes such as PBRM1, BAP1, SETD2, and KDM5C, implying that epigenetic dysfunction plays a role in the pathogenesis of this malignant disease [107, 108]. According to the study, widespread changes in DNA methylation can be detected in ccRCC and affect regions that develop the kidney genome [92]. Changes in the novel and prominent copy numbers in ccRCC samples are also seen in the large TCGA collection of ccRCC samples [109]. The analysis of the various methylated regions in the ccRCC revealed enrichment at Hairy-related transcription factor (HRT) binding sites [92]. Because HRT is the NOTCH signature route's mediator. Furthermore, recent research suggests that the NOCH blockade may be effective in a variety of adverse events [110]

There have been reports that NOTCH method components are activated in kidney cell cancer and that components such as DLL4 may have therapeutic efficacy in pre-clinical models [111]. However, little is known about the mechanisms that cause the NOTCH method to be activated in renal cell cancer. Recently, researchers examined the genetic and epigenetic abnormalities associated with the NOTCH approach in ccRCC and discovered that the ligands JAGGED1 and JAGGED2 were extremely prominent and associated with both genetic and epigenetic mutations. NOTCH activation has also been found to be widespread in large TCGA data sets. In vivo, transgenic NOTCH1 overexpression resulted in dysplastic and hyperproliferative tubes, demonstrating the carcinogenic role of this mechanism in RCC [112]. Finally, the clinical treatment inhibitor of the NOTCH LY-3039478 method led to an increase in survival in ccRCC xenografts, indicating this method as a treatment in the ccRCC [110]. The clinical trials (Reported in the United States National Institutes of Health (NIH) Library) of Epigenetic drugs with combinational therapies are in consideration [113]. The drugs belonging to HADCi class such as vorinostat [114], panobinostat [115], romidepsin [116], and belinostat [117] are reported to be in phase I and II clinical trials. DNMT inhibition drugs (azacytidine [118], oligonucleotide MG98 [119]) are reported in phase I/II clinical trials. Other therapeutics such as miRNA MRX34 [119], oligonucleotide GTI-2040 [120], and oligonucleotide oblimersen [121] are also in trials (Table 3).

**Table 3:** Ongoing clinical trials HDAC: histone deacetylase; DNMT: DNA methyltransferase; IFN: interferon; IL: interleukin

Epigenetic Drug	Combined Therapy	Phase	Trial Registry (NIH Library)	References
HDAC Inhibition				
Vorinostat	-	II	NCT00278395	(113)
	Isotretinoin	I/II	NCT00324740	
	Bevacizumab	I/II	NCT00324870	
Panobinostat	Sorafenib	I	NCT01005797	(114)
	-	II	NCT00550277	
	Everolimus	I/II	NCT01582009	
	IL-2	I/II	NCT01038778	
	IL-2	I/II	NCT03501381	
	Atezolizumab plus Bevacizumab	I/II	NCT03024437	
	Nivolumab plus Ipilimumab	II	NCT03552380	
Romidepsin	-	I	NCT01638533	(115)
	-	II	NCT00106613	
Belinostat	-	I	NCT00413075	(116)
DNMT Inhibition				
Azacytidine	IFN-α	I	NCT00217542	(117)
	Bevacizumab	I/II	NCT00934440	
	IFN-α	II	NCT00561912	
	Anti-PD-1	I/II	NCT02961101	
	MBG453	I	NCT02608268	
	Oxaliplatin	II	NCT04049344	
Oligonucleotide MG98	-	I/II	NCT00003890	(118)
Other Therapeutic Strategies				
miRNA MRX34	-	I	NCT01829971	(119)
Oligonucleotide GTI-2040	Capecitabine	I/II	NCT00056173	(119)
Oligonucleotide Oblimersen	IFN-α	II	NCT00059813	(120)

## Conclusions

The recent revolution in DNA methylation revolutionized the traditional view of gene function and its alteration as the primary cause of cancer and its metastases. Recent epigenetic advances have revealed that genome packaging is just as important as the genome in regulating the fundamental cellular processes that cause cancer. A better understanding of these epigenetic changes in cancer will lead to better therapeutic modalities, which will improve patient morbidity and mortality. The combined approach of epigenetic therapy in addition to standard chemotherapy promises successful cancer treatment. We hope these additional therapeutic approaches may also help cancer stem cells that are unresponsive to standard chemotherapy and are more likely to develop early metastases. Understanding cancer stem cells and developing specific epigenetic drugs are essential to effectively reconstructing the abnormal cancer epigenome. Epigenetic modification patterns linked to cancer development and progression have the potential to be clinically useful. The development of DNA methylation markers may be useful for early cancer detection, cancer diagnosis, and cancer prognosis prediction. Recent epigenomic advances allow for high-precision mapping of methylation/acetylation status and miRNA levels in the genome, which can aid in the identification of biomarkers for various diseases. Understanding the molecular events that initiate and maintain epigenetic gene silencing could lead to the development of clinical cancer prevention and treatment strategies.
